# Frequency of dysphagia among patients submitted to a rheumatology department: a cross-sectional analysis based on the EAT-10 questionnaire

**DOI:** 10.1007/s00296-025-05952-x

**Published:** 2025-08-12

**Authors:** Eleni C. Pardali, Katerina-Maria Kontouli, Anastasios Manolakis, Paraskevi Detopoulou, Konstantinos Argyriou, Irene Α. Tsakmaki, Theodora Simopoulou, Christina G. Katsiari, Dimitrios G. Goulis, Andreas Kapsoritakis, Dimitrios P. Bogdanos, Maria G. Grammatikopoulou

**Affiliations:** 1 Immunonutrition Unit, Department of Rheumatology and Clinical Immunology, Faculty of Medicine, School of Health Sciences, University of Thessaly, Biopolis campus, Larissa, Greece; 2 Department of Primary Education, School of Education, University of Ioannina, Ioannina, Greece; 3 Department of Gastroenterology, General University Hospital of Larissa, Larissa, Greece; 4 Department of Nutritional Sciences and Dietetics, University of the Peloponnese, Kalamata, Greece; 5 Unit of Reproductive Endocrinology, 1st Department of Obstetrics and Gynecology, Medical School, Aristotle University of Thessaloniki, Papageorgiou General Hospital, Thessaloniki, Greece

**Keywords:** Deglutition disorders, Swallow, Gastrointestinal, Oropharyngeal, Scleroderma, Esophageal dysphagia, Rheumatoid arthritis

## Abstract

Symptoms related to difficulty in swallowing and bolus transfer from the oral towards the gastric cavity have been collectively termed “dysphagia”. The etiology, gravity and complications of dysphagia warrant a rigorous, often multidisciplinary diagnostic evaluation and individualized management. The present study aimed to evaluate the prevalence of dysphagia among patients with rheumatic and musculoskeletal diseases (RMDs) and identify associated risk factors. This cross-sectional study included 340 consecutive patients diagnosed with RMDs from the Department of Rheumatology and Clinical Immunology situated at the Larissa University General Hospital. Dysphagia was assessed using the Eating Assessment Tool-10 (EAT-10). Univariate and multivariate analyses were performed to explore the associations between dysphagia, specific rheumatic diagnoses, and other contributing clinical and demographic factors. Based on the EAT-10, dysphagia was identified in 35% of the patients, with the most commonly reported issues being difficulty in swallowing solid foods and the sensation of food sticking in the throat. Patients with systemic sclerosis, primary Sjögren’s syndrome, and rheumatoid arthritis were significantly more likely to develop swallowing difficulties. Furthermore, low serum albumin levels, increasing age, and the presence of gastroesophageal reflux were identified as independent predictors of dysphagia. Both overweight/obesity and a diagnosis of axial spondyloarthritis were negatively associated with dysphagia. Age and years since diagnosis were positively correlated with an EAT-10 score ≥ 3, while body mass index and body fat percentage showed negative correlations. Swallowing dysfunction is prevalent among patients with RMDs, particularly in those with specific rheumatic diagnoses. Given its impact on nutritional status and overall quality of life, routine dysphagia screening in clinical settings is essential for the timely identification and management of the condition.

**Supplementary Information:**

The online version contains supplementary material available at 10.1007/s00296-025-05952-x.

## Introduction

Rheumatic and musculoskeletal diseases (RMDs) comprise a group of disorders that impact a significant portion of the global population [1]. Approximately 1.31 billion people worldwide are affected by musculoskeletal disorders, with an estimated 19.97 million individuals suffering from rheumatoid arthritis (RA) [1]. RMDs are linked to inflammation, although their exact cause remains unknown [2]. They present a range of symptoms, including joint pain, reduced physical activity, and gastrointestinal (GI) complaints [3]. RMDs like systemic sclerosis (SSc) [4, 5] and primary Sjögren’s syndrome (pSS) [6] can trigger GI symptoms through the induction of intestinal fibrosis, vascular remodeling, and alterations in intestinal permeability, secretory and autonomous nerve function.

One of the most debilitating GI symptoms in this context is dysphagia, or difficulty swallowing. Swallowing or deglutition, is a complex multi-step reflex process involving multiple levels of the central nervous system, coordinating around 50 pairs of cranial muscles [7]. Dysphagia, or difficulty swallowing, occurs when this complex process is disrupted and may manifest as solid-bolus, liquid-bolus dysphagia or both [7]. It is categorized as either pharyngeal –due to issues like weak pharyngeal contraction or obstruction–, or esophageal, linked to esophageal obstruction or hypomotility [4, 8]. This condition is linked with psychological, emotional, and social challenges [9], prolonged length of hospital stay (LOS), increased healthcare costs [10], morbidity and mortality [11].

Dysphagia can manifest in patients with RMDs [12] – a condition that has recently been termed autoimmune dysphagia [13, 14] – although research on this topic remains limited. Previous studies have reported dysphagia in patients with SSc [15, 16], pSS [6], ankylosing spondylitis [17], myositis [18], as well as, those with fibromyalgia syndrome (FMS) [19–21], which occurs mainly as a result of cricoarytenoid joint dysfunction [22], combined with oropharyngeal and esophageal dysfunction, painful mastication, and xerostomia [8, 13]. In RMDs, GI manifestations like dysphagia are often overshadowed by musculoskeletal symptoms [8], yet they can significantly impair daily [15] and social functioning, and quality of life [23] of patients. Swallowing difficulties may lead to emotional distress [24], and may in turn increase the risk of malnutrition [25], sarcopenia [26], and cachexia [27]. Due to the variety in assessment methods and the diversity of clinical symptoms, significant uncertainty remains regarding the true prevalence and risk factors of dysphagia in RMDs. Reliable epidemiological data on RMDs are essential to understand dysphagia’s burden in full extent and guide future research.

### Objectives

The aim of this study was to identify patients with RMDs with possible dysphagia, using the validated Eating Assessment Tool-10 (EAT-10) [28], and assess key factors that contribute to an increased risk of dysphagia.

## Materials and methods

### Study design and sample

The present single-center, cross-sectional study was conducted at the Department of Rheumatology and Clinical Immunology of the University General Hospital of Larissa. A total of 340 consecutive patients were recruited between February 2023 and January 2025 during their visits to inpatient and outpatient clinic, or for their scheduled intravenous immunotherapy sessions. The sample size was not calculated since the data corresponded to a specific hospital in a defined region. Inclusion criteria involved (i) patients diagnosed with at least one RMD, (ii) able to communicate effortlessly in the Greek language. There were no exclusion criteria except for concomitant cancer diagnosis, pregnancy and age younger than 18 years; all consecutive patients meeting the inclusion criteria were recruited for the study. No upper age limit was applied. Patients’ characteristics are detailed in Table 1. For easier classification, RA and juvenile idiopathic arthritis (JIA) were grouped together under the term “arthritis”. Similarly, diagnoses including IgA vasculitis, eosinophilic granulomatosis with polyangiitis, polyangiitis, granulomatosis with polyangiitis, Buerger’s disease, Cogan syndrome, and giant cell arteritis (GCA) were grouped as “vasculitides”. Dermatomyositis and other idiopathic inflammatory myopathies were grouped under the term ‘myositides’. Tumor necrosis factor receptor-associated periodic syndrome (TRAPS) and familial Mediterranean fever (FMF) were classified as “autoinflammatory syndromes”. The sample also included diagnoses such as systemic lupus erythematosus (SLE), axial spondyloarthritis (SpA), SSc, psoriatic arthritis (PsA), pSS, gout, antiphospholipid syndrome (APL), FMS, sarcoidosis, reactive arthritis (REA), and retroperitoneal fibrosis (RPF) (Table 1). Blood parameters for each patient were obtained from the medical records. The study was approved by the Larissa Hospital Scientific Board (30/3rd /20-02-2025), and all participants provided informed consent.

**Table 1:** Characteristics of the study population (*N* = 340)

Variables	Categories and units	Values
Sex	Women (*n*, %)	245 (72.1)
	Men (*n*, %)	95 (27.9)
Age (age)	Women (*n*, %)	60.32 ± 14.5^∝^
	Men (*n*, %)	60.34 ± 17.3^∝^
Education	Primary school (*n*, %)	151 (44.7)
	Middle school (*n*, %)	111 (32.8)
	Tertiary (*n*, %)	71 (21.0)
	Postgraduate (*n*, %)	5 (1.5)
Employment	Full-time (*n*, %)	85 (25.1)
	Part-time (*n*, %)	17 (5.0)
	Unemployed (*n*, %)	59 (17.4)
	Retired (*n*, %)	151 (44.5)
	Ill-health retirement (*n*, %)	27 (8.0)
Ethnicity	Caucasian (*n*, %)	327 (96.8)
	Roma (*n*, %)	9 (2.7)
	Latino (*n*, %)	1 (0.3)
	American (*n*, %)	1 (0.3)
Current smokers	(*n*, %)	67 (19.8)
BMI (kg/m^2^)		27.7 ± 6.2^∝^
Weight status	Underweight (*n*, %)	13 (3.9)
	Normoweight (*n*, %)	98 (29.1)
	Overweight-Obese (*n*, %)	226 (67.1)
Recruitment	Inpatient (*n*, %)	160 (47.3)
	IV immunotherapy (*n*, %)	133 (39.3)
	Outpatient (*n*, %)	45 (13.3)
GERD	(*n*, %)	96 (28.4)
CRP (mg/dL)		5.8 ± 29.8^∝^
Albumin (g/dL)		3.92 ± 1.15^∝^
ESR (mm/h)		43.65 ± 36.4^∝^
On Corticosteroids	(*n*, %)	137 (40.3)
RMDs	Arthritis (RA, JIA) (*n*, %)	124 (36.5)
	SLE (*n*, %)	50 (14.7)
	Vasculitides (IgA vasculitis, eosinophilic granulomatosis with polyangiitis, granulomatosis with polyangiitis, polyangiitis, Behçet, Buerger’s, Cogan, GCA, Takayasu) (*n*, %)	45 (13.2)
	PsA (*n*, %)	29 (8.5)
	Multiple RMD diagnoses (*n*, %)	28 (8.2)
	Myositides (*n*, %)	28 (8.2)
	SSc (*n*, %)	21 (6.2)
	SpA (*n*, %)	21 (6.2)
	Undiagnosed yet (*n*, %)	15 (4.4)
	pSS (*n*, %)	14 (4.1)
	Gout (*n*, %)	7 (2.1)
	APL (*n*, %)	5 (1.5)
	FMS (*n*, %)	4 (1.2)
	Autoinflammatory syndromes (TRAPS, FMF) (*n*, %)	3 (0.9)
	Sarcoidosis (*n*, %)	3 (0.9)
	REA (*n*, %)	1 (0.3)
	RPF (*n*, %)	1 (0.3)

*APL* antiphospholipid antibody syndrome, *AS*: ankylosing spondylitis; *BMI*: body mass index; *CRP*: C-reactive protein; *dL*: deciliter; *ESR*: erythrocyte sedimentation rate; *FMF*: familial Mediterranean fever; *FMS*: fibromyalgia syndrome; *g*: grams; *GCA*: giant cell arteritis; *hr*: hour; *IV*: intravenous; *JIA*: juvenile idiopathic arthritis; *mg*: milligrams; *mm*: millimeters; *PsA*: psoriatic arthritis; *pSS*: primary Sjögren’s syndrome; *RA*: rheumatoid arthritis; *REA*: reactive arthritis; *RMDs*: rheumatic musculoskeletal diseases; *RPF*: retroperitoneal fibrosis; *SD*: standard deviation; *SLE*: systemic lupus erythematosus; *SpA*: spondyloarthritis; *SSc*: systemic sclerosis; *TRAPS*: tumor necrosis factor receptor-associated periodic syndrome.^∝^ Mean ± SD

### Data collection

The anthropometric measurements of all patients were recorded according to standardized procedures by experienced dietitians. Body weight and height were measured using a digital floor scale (Kern MPE 200 K-1PEM, Kern, Germany) and a stadiometer (Seca 220, Hamburg, Germany), respectively. Body mass index (BMI) was calculated for all patients, and they were categorized into three subgroups: underweight (BMI < 18.5 kg/m^2^), normoweight (BMI ≥ 18.5 kg/m^2^ and BMI < 25 kg/m^2^), and overweight/obesity (BMI ≥ 25 kg/m^2^).

To assess body fat (BF) percentage (of body mass), skinfold thickness was measured at four specific anatomical sites: biceps, triceps, subscapular, and suprailiac. A Slimguide set of calipers (Creative Health, USA) was used for all measurements, which were taken on the right side of each participant’s body. Each site was measured twice, and the median value was used for the final calculations. The Jackson and Pollock Eqs. [29, 30] provided the formula for calculating BF percentage, as a proportion of total body weight.

### Dysphagia assessment

Dysphagia was assessed using the validated Greek version [31] of the EAT-10 questionnaire [28]. This patient-reported Likert scale tool consists of 10 items, each corresponding to 5 levels of difficulty, ranging from 0 (“no problem”) to 4 (“severe problem”). The total score ranges from 0 to 40, and a score ≥ 3 is considered abnormal, indicative of possible dysphagia [28].

### Statistical analyses

The continuous variables were expressed as means along with their standard deviations (SD), whereas the categorical variables were presented as frequencies and corresponding percentages. To investigate the associations between sarcopenia assessment and various covariates—including age, weight status, smoking status, gastroesophageal reflux (GERD), albumin, C-reactive protein (CRP), erythrocyte sedimentation rate (ESR), current corticosteroid use, and the presence of diseases such as RA, SLE, vasculitides, SpA, myositides, SSc, PsA, and pSS—we initially conducted a univariate logistic regression analysis. Based on the variables that showed statistical significance in the univariate analysis, we proceeded with a multivariate logistic regression model to account for potential confounders. To refine the model, we applied backward elimination, ensuring that only the most relevant predictors were retained. Additionally, we developed an alternative multivariate model incorporating CRP, albumin, years from the initial diagnosis, and BMI. Odds ratios (ORs) and their corresponding 95% confidence intervals (CIs) were calculated to quantify these associations. A complete-case analysis was performed to mitigate any potential bias arising from missing data. Statistical significance was determined using a threshold of *p* < 0.05, and all analyses were conducted using R Studio (version 4.4.1) [32].

## Results

### Dysphagia and EAT-10 questionnaire

**Fig. 1:**
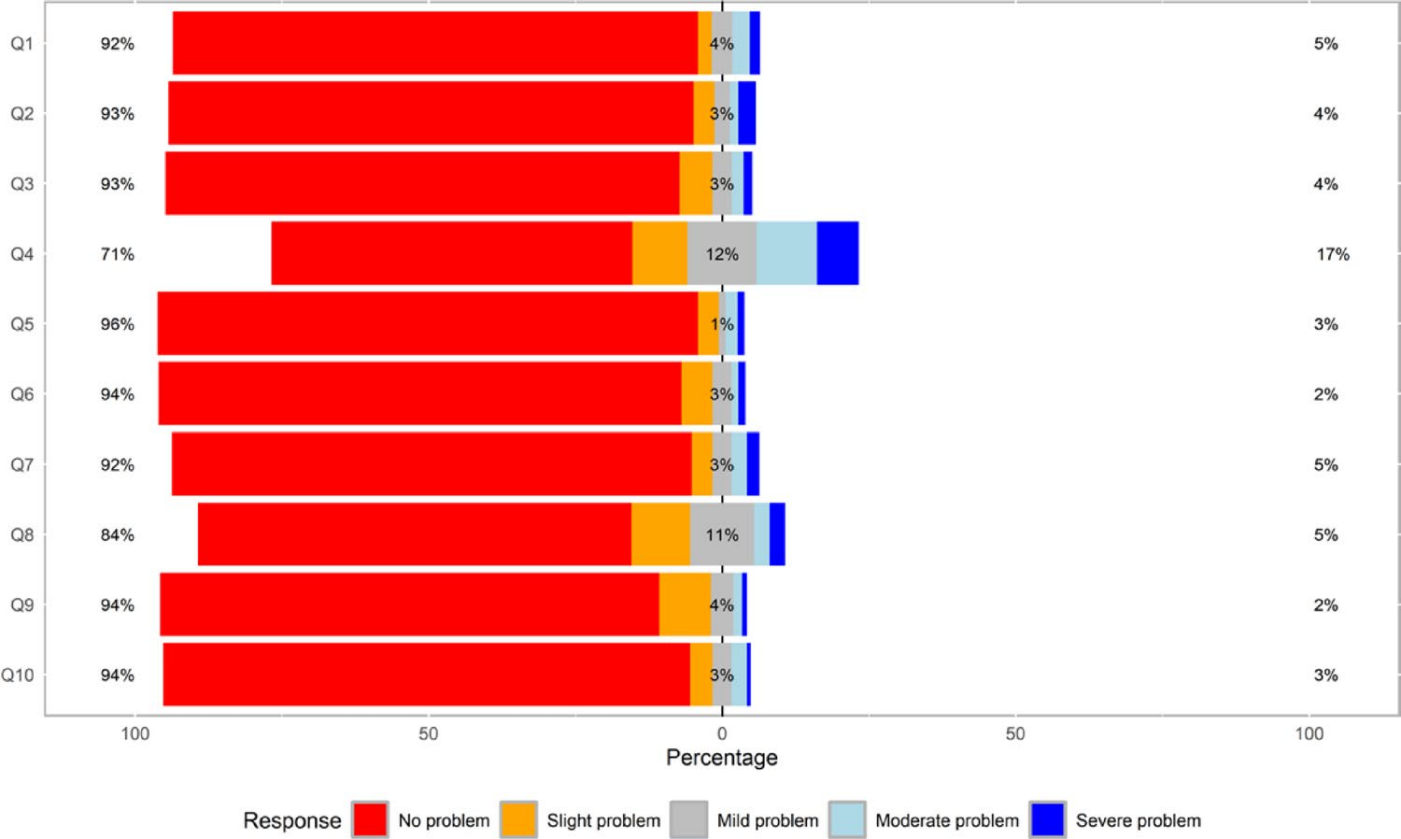
Distribution of the EAT-10 questionnaire responses among patients with RMDs. *EAT-10* eating assessment tool-10; Q1: My swallowing problem has caused me to lose weight; Q2: My swallowing problem interferes with my ability to go out for meals; Q3: Swallowing liquids takes extra effort; Q4: Swallowing solids takes extra effort; Q5: Swallowing pills takes extra effort; Q6: Swallowing is painful; Q7: The pleasure of eating is affected by my swallowing; Q8: When I swallow food sticks in my throat; Q9: I cough when I eat; Q10: Swallowing is stressful; RMDs: rheumatic and musculoskeletal diseases

In the total sample, based on the EAT-10, dysphagia was present in 120 patients (35%). Additionally, 13.5% (*n* = 46) of the patients exhibited mild-to-moderate symptoms (0 > EAT-10 ≤ 2), including 8.5% (*n* = 29) with an EAT-10 score of 1 and 5% (*n* = 17) with an EAT-10 score of 2, without reaching the screening threshold for dysphagia. Among those with possible dysphagia (EAT-10≥3), 17% of the participants reported severe problems with swallowing solids, while 12% experienced mild difficulty. Regarding food sticking in the throat, 5% reported having severe difficulties, and 11% reported mild concerns. Additionally, 5% of patients indicated that swallowing difficulties had led to weight loss and affected eating enjoyment, while 4% reported mild issues with coughing while eating (Fig. 1). Table 2 displays the prevalence of dysphagia among patients with different RMD diagnoses, as assessed using the EAT-10 tool.

**Table 2:** Prevalence of dysphagia assessed by the EAT-10 in patients with RMDs (Ν = 340)

Diagnosis	Total (*n*)	Patients with dysphagia (*n*)	Dysphagia prevalence (%)
RA	116	47	40.5
JIA	8	1	12.5
SLE	50	13	26
Vasculitides (IgA vasculitis, eosinophilic granulomatosis with polyangiitis, granulomatosis with polyangiitis, polyangiitis, Behçet, Buerger’s, Cogan, GCA, Takayasu)	45	16	35.6
PsA	29	9	31
Multiple RMD diagnoses	28	14	50
Myositides	28	12	42.9
SSc	21	16	76.2
SpA	21	2	9.5
Undiagnosed yet	15	1	6.7
pSS	14	11	78.6
Gout	7	3	42.9

The table does not include patient groups with less than six patients in each diagnosis. * AS* ankylosing spondylitis, * GCA* giant cell arteritis, * JIA* juvenile idiopathic arthritis;* PsA* psoriatic arthritis, * pSS* primary Sjögren’s syndrome,* RA* rheumatoid arthritis, * RMDs* rheumatic musculoskeletal diseases, * SLE* systemic lupus erythematosus,* SpA* spondyloarthritis,* SSc* systemic sclerosis

### Univariate and multivariate logistic regression analyses

Significant predictors of dysphagia (Table 3) were age (OR: 1.01, 95% CI: 1.00–1.01), patient-reported GERD (OR: 1.32, 95% CI: 1.18–1.48), and the diagnosis of SSc (OR: 6.18, 95% CI: 2.42–18.36), and pSS (OR: 6.53, 95% CI: 2.11–26.05). Overweight/obese status (OR: 0.88, 95% CI: 0.78–0.98) and diagnosis of SpA (OR: 0.22, 95% CI: 0.04–0.70) were both negatively linked to dysphagia. Using backward elimination, we performed a multivariate analysis including all variables of diseases that were used in the univariate analysis. SSc, pSS, and RA were significantly associated with dysphagia after adjusting for other diagnoses. In the second model of the multivariate analysis (Table 4), the only significant predictor of dysphagia was albumin (OR: 0.52, 95% CI: 0.29–0.91), while CRP concentrations, disease duration, and BMI did not appear to predict swallowing problems.

**Table 3:** Univariate and multivariate logistic regression analyses of dysphagia (Ν = 340)

Variables	Univariate		Multivariate	
	OR (95% CI)	*p* value	OR (95% CI)	*p* value
Age (age)	1.01 (1.00–1.01)	< 0.001*		
Underweight^ß^	1.19 (0.91–1.57)	0.21		
Overweight/obese^ß^	0.88 (0.78–0.98)	0.02*		
Smoking^#^	0.95 (0.83–1.08)	0.44		
GERD^#^	1.32 (1.18–1.48)	< 0.001*		
Albumin (g/dL)	1.01 (0.96–1.07)	0.69		
CRP (mg/dL)	1.00 (1.00–1.00)	0.25		
ESR (mm/h)	1.00 (1.00–1.00)	0.19		
Corticosteroids use^#^	1.05 (0.94–1.16)	0.4		
RA^#^	1.06 (0.95–1.17)	0.32	1.13 (1.01–1.27)	0.036*
SLE^#^	0.90 (0.78–1.04)	0.14		
Vasculitides^#^	1.00 (0.86–1.17)	0.97	1.11 (0.95–1.30)	0.174
SpA^#^	0.22 (0.04–0.70)	0.008*	0.84 (0.68–1.04)	0.104
Myositides^#^	1.43 (0.65–3.07)	0.37	1.18 (0.98–1.42)	0.084
SSc^#^	6.18 (2.42–18.36)	< 0.001*	1.66 (1.35–2.05)	< 0.001*
PsA^#^	0.83 (0.36–1.82)	0.65		
pSS^#^	6.53 (2.11–26.05)	< 0.001*	1.67 (1.31–2.14)	< 0.001*

*CI* confidence intervals,* CRP* C-reactive protein, *dL* deciliter, *ESR* erythrocyte sedimentation rate, *g* grams,* GERD* gastroesophageal reflux,* h* hour, *mg* milligrams, *mm* millimeters, *OR* odds ratio, *PsA* psoriatic arthritis,* RA* rheumatoid arthritis, *pSS* primary Sjögren’s syndrome, *SLE* systemic lupus erythematosus, *SpA* spondyloarthritis,* SSc* systemic sclerosis. β in comparison to normoweight, # binary data (yes/no), * p < 0.05

**Table 4:** Multivariate logistic regression analysis assessing the association between dysphagia and albumin, CRP, years from the initial diagnosis, and BMI

Variables	Multivariate	
	OR (95% CI)	*p* value
Albumin (g/dL)	0.52 (0.29–0.91)	0.02*
CRP (mg/dL)	0.98 (0.95–1.02)	0.46
Years from the initial diagnosis	1.02 (1.00–1.05)	0.09
BMI (kg/m^2^)	0.98 (0.94–1.04)	0.65

*BMI* body mass index,* CI*: confidence intervals,* CRP* C-reactive protein,* dL* deciliter,* g* grams,* kg* kilograms,* m* meters,* mg* milligrams,* OR* odds ratio. * p < 0.05

### Factors associated with dysphagia

**Fig. 2:**
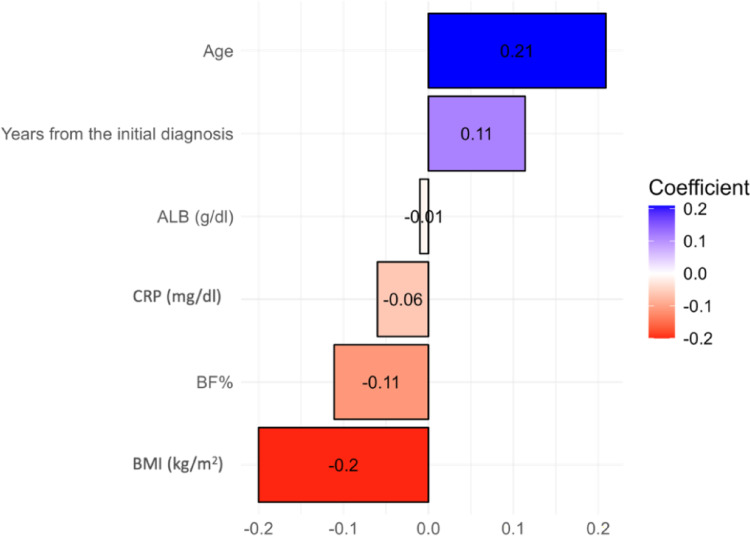
Linear correlations between several variables and the EAT-10 questionnaire. *ALB* albumin,* BF%* body fat percentage,* BMI* body mass index,* CRP* C-reactive protein,* dl* deciliter,* g* grams,* kg* kilograms,* m* meters,* mg* milligrams,* EAT-10* eating assessment tool-10

The heatmap chart (Fig. 2) presents the correlation between the EAT-10 questionnaire and various clinical and demographic variables. Specifically, age (*r* = 0.21) and years from the initial diagnosis (*r* = 0.11), showed moderate correlations with an EAT-10 score ≥ 3. In contrast, BMI (*r* = -0.2), and BF (% of body weight) (*r* = -0.11), CRP (*r* = -0.06) and albumin (*r* = -0.01) concentrations exhibited negative correlations.

## Discussion

The present cross-sectional study revealed the presence of possible dysphagia based on the EAT-10 questionnaire, in patients with RMDs. Notably, individuals diagnosed with SSc, pSS and RA had a significantly greater risk for developing dysphagia, while the likelihood of experiencing this condition increased progressively with advancing age.

### Utilization of the EAT-10 questionnaire for dysphagia assessment in RMDs

Of the 340 patients included in the study, 120 exhibited dysphagia (i.e. about 35%), as indicated by the EAT-10 questionnaire, with the majority of those reporting difficulty in swallowing solid foods and food sticking in the throat. Additionally, people with dysphagia reported difficulty in maintaining their current weight, enjoying food, and socializing, and some reported that swallowing caused anxiety, adding another stressor to their current situation. Indeed, psychological distress may exacerbate perceived swallowing difficulties, while dysphagia itself may contribute to increased anxiety, suggesting a reciprocal relationship between dysphagia and anxiety [33].

To our knowledge, only four studies have used this specific tool to assess dysphagia in a similar patient population. The first study examined patients with ankylosing spondylarthritis and found a greater incidence of dysphagia in this group compared to healthy controls [17]. Two additional studies focused on patients with FMS [19, 20] and SSc [15] and reported dysphagia in 65.21% and 52% of the samples, respectively. Finally, in the study by Gilheaney et al. [21]. , 73% of patients with FMS experienced dysphagia, with the majority experiencing food sticking in the throat and difficulty swallowing pills and solids. Despite these findings, we were unable to specifically evaluate FMS-related dysphagia in our cohort due to the small sample of patients with an FMS diagnosis. Overall, autoimmune dysphagia has been reported in up to 73% of patients, with the prevalence varying, depending on the underlying disease and the assessment method used [13, 21]. Previous research has suggested reducing the EAT-10 cut-off value from 3 to 2, a change aimed at improving sensitivity, while maintaining specificity [34]. However, this adjustment has not yet been validated in the context of RMDs and requires further investigation to determine its applicability and clinical relevance in this patient population.

### Determinants of dysphagia in RMDs

In the current sample, patient-reported GERD, advancing age, weight status, and certain diagnoses such as SSc, pSS, and SpA were associated with the development of dysphagia. GERD is a highly prevalent GI disorder that affects a great proportion of individuals with RMDs, a relationship that has been further supported by genetic studies [35, 36]. In addition, patients with SSc may be at increased risk for developing GERD due to hypomobility of the esophageal body, mainly affecting the smooth muscle fibers and reduced resting pressure of the lower esophageal sphincter [4]. Within this cohort, patients who reported GERD exhibited a 32% increased likelihood of experiencing dysphagia. Heartburn and regurgitation, common in GERD, are associated with non-obstructive dysphagia due to upper esophageal sphincter dysfunction, acid hypersensitivity, and esophageal dysmotility [37]. Recent findings by Reddy et al. [38]. indicate that the majority of patients with RMDs have a unique esophageal motility disorder that is primarily characterized by achalasia, hypo-contractile esophagus, and esophagogastric junction outflow obstruction. Elderly people are at increased risk of developing dysphagia, mainly due to age-related changes in swallowing function, both motor and sensorimotor [39]. Herein, we observed that for each additional year of age, the odds of dysphagia increased by 1% in the sample.

Interestingly, overweight and obesity status appeared to exert a protective effect against dysphagia, with patients in this weight status categories showing a 12% reduced risk of experiencing swallowing difficulties. To our knowledge, no previous studies have reported similar findings. In contrast, the existing literature [40] suggests that overweight status and obesity are linked to esophageal dysfunction, increased esophageal acid exposure, and other GI manifestations, all of which are known to contribute to dysphagia. Furthermore, underweight status has previously been associated with an increased risk of dysphagia [41]—a relationship not observed herein. However, after adjustment for co-founders, BMI did not appear to influence the odds of dysphagia. The unexpected findings regarding BMI and dysphagia risk may be attributed to an imbalance in the distribution of patients across BMI categories or the influence of confounding factors, such as medication use, lifestyle habits, or underlying comorbidities. Furthermore, it is highly likely that the increased BMI may actually be the result of lack of dysphagia, leading to an increased dietary intake and greater BMI. Further research is warranted to explore these potential explanations and to clarify the complex relationship between body weight and dysphagia.

In terms of rheumatic disease diagnoses, patients with SSc had more than a sixfold increased risk of developing dysphagia, while those with pSS had a 6.53-fold higher risk. Previous research has highlighted the significant GI discomfort experienced by individuals with these diagnoses [5, 6, 15]. SSc is a systemic autoimmune disorder characterized by vascular dysfunction, progressive fibrosis of the skin and internal organs, including involvement of the GI tract. In addition to cutaneous manifestations, patients often suffer from microstomia, oral ulcers, and esophageal dysfunction, all of which contribute to swallowing difficulties [15]. On the other hand, pSS is primarily defined by chronic inflammation and dysfunction of the salivary and lacrimal glands, resulting in severe oral and ocular dryness, as well as reduced secretions within the GI tract [42]. This persistent dryness significantly impairs bolus formation and swallowing, further predisposing patients to dysphagia [6].

In contrast, people with SpA appeared to be less likely to experience dysphagia. Given that SpA primarily affects the spine, with potential manifestations in the GI tract [43], this observation was particularly intriguing. Although the literature on this topic is limited, several case reports have highlighted the potential association between dysphagia and cervical spine involvement in SpA patients [44, 45], while the prevalence of dysphagia has been shown to be greater in individuals with the condition compared to those without [17]. Nevertheless, the backward elimination multivariate analysis showed no significant association between SpA and dysphagia.

Interestingly, the same model highlighted that patients with RA had a 13% increased risk of dysphagia. RA-related cricoarytenoid arthritis can affect the upper airway, while symptoms like xerostomia, impaired mastication, and masticatory pain have also been documented to contribute to dysphagia in this population [46]. Nonetheless, myositis, vasculitides, PsA, and SLE lacked association with dysphagia. Specifically in myositis, dysphagia is associated with several adverse events, most commonly aspiration [18]. Limited evidence, primarily from case reports, suggests an association between SLE and vasculitides with GI discomfort and dysphagia. Reported cases have included esophageal and oropharyngeal dysphagia, respectively, as well as the involvement of the secondary neurological syndromes [47–49]. However, more studies need to be conducted to elucidate this relationship. Furthermore, to our knowledge, no current research item has investigated a possible relationship between PsA and dysphagia, leaving this as an unexplored area for future research.

### The interplay between dysphagia, malnutrition, and sarcopenia

Low serum albumin levels have been previously linked to impaired swallowing and an elevated risk of mortality in patients with dysphagia, particularly among older individuals [50, 51]. In the present study, albumin exhibited a modest negative correlation with dysphagia, and lower albumin levels were found to be independently associated with swallowing difficulties, after controlling for possible confounders.

A bidirectional relationship exists between dysphagia and malnutrition, whereby swallowing difficulties can result in inadequate nutrient intake, and nutritional deficiencies can further exacerbate the patient’s condition by impairing GI function and contributing to worsening dysphagia [52, 53]. This cycle can ultimately result in sarcopenia, as insufficient energy and macronutrient intake deprive the body of essential resources, leading to muscle wasting and frailty that is independent of aging [54]. Research in patients with SSc has revealed that malnutrition may be a useful predictor of GI involvement in routine clinical practice [16]. The strong interplay between dysphagia, malnutrition, and sarcopenia is of paramount importance, particularly in patients with RMDs, highlighting the need for timely and systematic hospital-based screening and management, as emphasized in previous studies [25, 26]. A recent study highlighted significant gaps in the knowledge of healthcare professionals regarding enteral and parenteral nutrition, with over 80% of participants reporting inadequate training in these domains [55]. With respect to the assessment of dysphagia, only 7% of clinicians relied on patient-reported symptoms, 19.2% employed direct observation of eating or drinking, and 42.3% utilized a combination of both methods [55]. These findings underscore the urgent need for enhanced educational and training initiatives, particularly among healthcare professionals treating high-risk patient populations, to address the critical aspects of dysphagia assessment and nutritional management.

### Management strategies for dysphagia

Effective swallowing management plays a crucial role in maintaining both nutritional status and improving overall disease outcomes [12]. Proper swallowing function ensures the activation and strengthening of GI tract muscles, minimizes the risk of aspiration, and enhances nutrient absorption, thereby reducing the likelihood of malnutrition and sarcopenia. A multidisciplinary approach is necessary to address the challenges associated with dysphagia, involving speech-language pathologists who focus on behavioral strategies to mitigate choking risks and alleviate aspiration-related anxiety, along with nutritionists who optimize dietary adjustments for improved swallowing performance [39]. Several therapeutic strategies have been developed to enhance swallowing function. Postural adjustments can significantly affect the velocity, direction, and clearance of a bolus or liquid, facilitating safer swallowing. Patients suffering from upper oropharyngeal and esophageal dysfunction may benefit from targeted swallowing maneuvers that enhance upper esophageal sphincter function and promote bolus passage [39]. In patients whose dysphagia results from autoimmune-mediated muscle weakness, individualized swallowing interventions may help prevent GERD and the formation of esophageal diverticula [8]. With regard to oesophageal strictures, a common therapeutic approach is dilation, which can be achieved by techniques such as through-the-scope balloon dilation or bougienage [56]. In cases of severe or refractory symptoms, these interventions may help restore esophageal patency, alleviate dysphagia, and improve nutritional intake and overall quality of life.

In addition to these therapeutic measures, dietary modifications are also critical in the management of dysphagia. The use of thick liquids has been demonstrated to assist in regulating the flow of the bolus, thereby enhancing the control of mechanical swallowing. Furthermore, the modification of the texture of solid foods, such as making them more cohesive, homogenized, or moisture-enhanced, has been shown to reduce the risk of aspiration and facilitate safer consumption [39, 57]. In this context, the International Dysphagia Diet Standardisation Initiative (IDDSI) serves as a globally unified framework that standardizes the classification of texture-modified foods and thickened liquids, ensuring a shared understanding between clinicians and patients regarding dysphagia diets [58]. Such modifications may benefit patients with temporomandibular disorders, such as those observed in RA; however, they should be supervised by a qualified expert to ensure that nutritional adequacy is maintained [59]. On the other hand, individuals afflicted with esophageal dysmotility are advised to consume small, thoroughly chewed food bolus, abstain from dry foods, ensure sufficient fluid intake alongside solid meals, and refrain from assuming a prone position for an extended duration after meal [39].

In addition to behavioral and dietary interventions, pharmacological approaches hold promise in the management of swallowing disorders. Immunotherapy with agents such as methylprednisolone, methotrexate, tacrolimus, mycophenolate, cyclophosphamide, rituximab, and hydroxychloroquine, as well as high-dose intravenous immunoglobulin, have demonstrated therapeutic effects in selected patients [60]. Treatment with proton pump inhibitors (PPIs) is also known as a practical approach to managing dysphagia [61]. Additionally, prokinetic agents have demonstrated promising results in patients with SSc experiencing dysphagia [62]. Proper patient selection and careful procedural management are essential for minimizing the risk of complications, such as perforation or recurrent strictures.

The impact of dysphagia is critical in many aspects. Dysphagia has been demonstrated to be independently associated with increased LOS and healthcare costs, and it affects both patient-related expenses and organizational burdens, such as staffing, feeding protocols, and discharge planning [10]. There is currently no standardized approach to dysphagia management, which underscores the need for clinical guidelines and integrated multidisciplinary care. Future research and policy could build upon these findings to establish screening protocols for dysphagia in both acute care and primary care settings.

### Limitations

The present study employs a cross-sectional design, precluding the establishment of causal relationships. However, it allows for the identification of factors associated with dysphagia in RMDs, including demographic characteristics and disease-related outcomes. Additionally, the assessment of dysphagia was not performed using objective measures of videofluoroscopy and fiberoptic endoscopy, or high-resolution esophageal manometry (HRM) and esophagogastroduodenoscopy (EGD), but rather using the validated EAT-10 questionnaire. Consequently, the precision of the findings may be limited. Furthermore, we did not collect data on esophageal amyloidosis secondary to RA mimicking achalasia, nor on severe esophagitis, Barrett’s esophagus, and strictures such as Schatzki rings, which are commonly observed in patients with SSc. These factors could serve as potential confounders.

In addition to that, the EAT-10 assessment does not distinguish between oropharyngeal and esophageal dysphagia. While it serves as a valuable, cost-effective tool for identifying possible dysphagia, it lacks the specificity needed for a comprehensive diagnosis. Therefore, further evaluation is essential to accurately determine the patient’s condition and develop a personalized care and treatment plan. Moreover, the absence of a control group limits the potential for comparative analyses that could yield valuable insights. Additionally, certain disease subcategories included a limited number of patients, which restricted further analyses to prevent a loss of statistical power.

## Conclusion

The current study identified swallowing problems among patients suffering from RMDs. Although the study design does not allow for the determination of cause-and-effect relationships, and the study sample cannot provide details about specific conditions, further research is needed to link swallowing problems and their outcomes in this population. The present study showed that conditions such as SSc, pSS, and RA and factors such as advancing age, the presence of GERD, and low albumin levels contributed to an increased likelihood of developing dysphagia. Given its significant impact on health and disease progression, routine screening for dysphagia in hospital settings is essential for timely diagnosis and management.

## Supplementary Information

Below is the link to the electronic supplementary material.

Supplementary Material 1

Supplementary Material 2

Supplementary Material 3

Supplementary Material 4

Supplementary Material 5

Supplementary Material 6

Supplementary Material 7

Supplementary Material 8

Supplementary Material 9

Supplementary Material 10

Supplementary Material 11

## Data Availability

The datasets collected for this manuscript are accessible from the corresponding author upon reasonable request.
